# Development and validation of inventory tool to evaluate social accountability principles in case scenarios used in problem-based curriculum (Social accountability inventory for PBL)

**DOI:** 10.1080/10872981.2020.1847243

**Published:** 2020-11-17

**Authors:** Mohamed Elhassan Abdalla, Nihar Ranjan Dash, Sarra Shorbagi, Mohamed H. Taha

**Affiliations:** a College of Medicine and Medical Education Center, University of Sharjah, Sharjah, United Arab Emirates; b Department of Clinical Sciences, College of Medicine, University of Sharjah, Sharjah, United Arab Emirates; c Department of Community and Family Medicine and Behavioural Sciences, College of Medicine, University of Sharjah, Sharjah, United Arab Emirates

**Keywords:** Social accountability, curriculum, problem-based learning

## Abstract

Social accountability (SA) is an obligation for medical schools in meeting the priority health concerns of the communities they serve. To measure the integration of SA principles into medical curricula, suitable tools are needed. This study developed and validated an inventory to assess SA values within the existing case scenarios used in problem-based learning (PBL) curricula. The Delphi technique was employed to develop and validate the new inventory. The validation used expert opinion and calculated the content validity using content validity indices (CVIs). The initial draft (Draft 0) was formulated with 25 open-ended questions. Following expert evaluation, Draft 1 had 22 closed-ended questions and the mean ratings, according to the experts, were as follows: relevance, 3.33–4.83; importance (3.5–4.8); clarity (3.33–4.83); and simplicity (3:00–4.67). Draft 2 had 19 questions. After a further round of rating and analysis, a final draft was prepared, consisting of 17 items, with CVI scores ≥ 0.8 and 100% overall satisfaction. Using this inventory tool will help health professions schools to translate SA indicators into curricular activities by identifying the gaps in their PBL curricula. Deficiencies can be either in the type of case scenarios used or the triggers embedded in the individual case scenarios, subsequently leading to the development of PBL case scenarios that address real health social needs. A revision and rewriting of the problem case scenarios to incorporate SA will be the next step.

## Introduction

In the past decade, medical education leaders emphasised the concept of social accountability (SA) in medical education [1]. In 1995 the World Health Organization (WHO) released its document *Defining and Measuring the Social Accountability of Medical Schools*, which acknowledged a need for SA in medical education [2] and defined SA as:

The obligation of the medical schools to direct their education, research and/or service activities towards addressing the priority health concerns of the community, region, and/or nation they have the mandate to serve. Priority health concerns are to be jointly identified by governments, health care organisations, health professionals, and the public [2].

The WHO document proposed relevance, quality, cost-effectiveness, and equity as principles of SA of the medical programmes [2]. Medical schools can apply these principles when planning, delivering and evaluating the impact of their education, research, and service programmes.

Subsequently, the Global Consensus for Social Accountability of Medical Schools (GCSA) [3] and the Association of Faculties of Medicine of Canada [4] linked the missions of medical schools and training of health professionals with the health needs of people.

Furthermore, the World Federation for Medical Education (WFME) has incorporated SA into the updated standards for the accreditation of medical education programmes [5]. The Network Towards Unity for Health (Network–TUFH) adopted the Tunisia Declaration for SA in 2017, calling for more concerted action to make SA a reality through partnerships with stakeholders [6]. Another remarkable SA development has been the inclusion of related standards in the National Health Workforce Accounts Handbook, developed by the WHO, which was launched in 2017 [7]. All these initiatives have emphasised that medical schools should collaborate with relevant stakeholders to identify their priority health needs and expectations and address the short- and long-term benefits for both the community and the medical school when responding to these needs.

Despite increased awareness and understanding of SA, translation of the concept into actual practice in the curricula and functions of medical schools needs more work [8]. Societies and their health needs differ across the globe; therefore, every country must define the indicators of SA for its medical schools and incorporate these indicators into their curricula and other activities [9].

Around the world, some medical schools have already incorporated SA concepts into their curricula (e.g. inclusion of community service in the curriculum) and have reported progress and achievements [10,11]. These initiatives have provided a precedent for other schools to follow, but have not specified a systematic structure for measuring progress towards goal achievement [12], SA published actions mostly focused on defining the dimensions and indices of SA in medical schools [13,14].

Models and frameworks for assessing SA within entire medical schools were published internationally, including the social accountability grid published by the WHO, the CPU model invented by Boelen and Wollard, and the Training for Health Equity Network (THEnet) framework. Individual countries also published their frameworks for SA [9,15–18]. Despite mentioning the above models and frameworks, the literature lacks a feasible and reliable instrument for measuring the implementation of SA in educational content [19].

According to the GCSA, outcome-based education is a strategic direction for SA, and problem-based learning (PBL) and student-centred education strategies are suitable vehicles for implementing SA principles [3]; therefore, the current study aimed to develop and validate an inventory tool for examining the PBL case scenarios used in PBL curricula, to determine their compliance with SA principles. Using this inventory tool, medical educators can evaluate the incorporation of SA principles into their curricula, especially PBL medical curricula.

## Methods

This was a methodological study employing the Delphi technique to develop and validate an inventory for assessing the SA values integrated into the case scenarios of PBL medical curricula. Development of the inventory based on the four values of SA: relevance, equity, quality, and cost-effectiveness [2].

The authors developed Draft 0 of the inventory based on the discussion of the four SA values and their application to PBL case construction. Draft 0 contained 25 open-ended questions formulated according to the four SA values. In the first round of evaluation, Draft 0 was sent to four medical education experts, three of whom had been significantly contributed to the development of SA concept and had involved in the development of the GCSA, they also had experience with PBL curricula, the fourth expert is a professor in medical education who had worked extensively with PBL curricula and case construction. These experts were asked to give their opinion on the draft content and the applicability of the inventory items for analysing the SA values within PBL case scenarios. Their comments were used to improve the inventory and develop Draft 1.

Draft 1 contained 22 closed-ended questions (with rating scales and options) covering the four SA values typically explored in PBL case scenarios. For the second round of analysis, eight evaluators were selected as judges for the inventory validation. The criteria for choosing the judges were adapted from those suggested by Fehring [20] as follows [1]: significantly involved in SA, preferably having participated in the development of the GCSA (Five Evaluators) [2]; has expertise in medical education and/or PBL curricula (Six Evaluators) [3]; is working as a faculty member in a medical school adopting PBL strategies (Five Evaluators); and [4] has written publications relating to PBL case construction (Three Evaluators). Most of the evaluators were cross-represented in two or more of the above criteria (e.g. four of them fulfilling criteria 1 and 2, three fulfilling criteria 1 and 3, and two fulfilling criteria 1 and 4).

**Figure 1.**
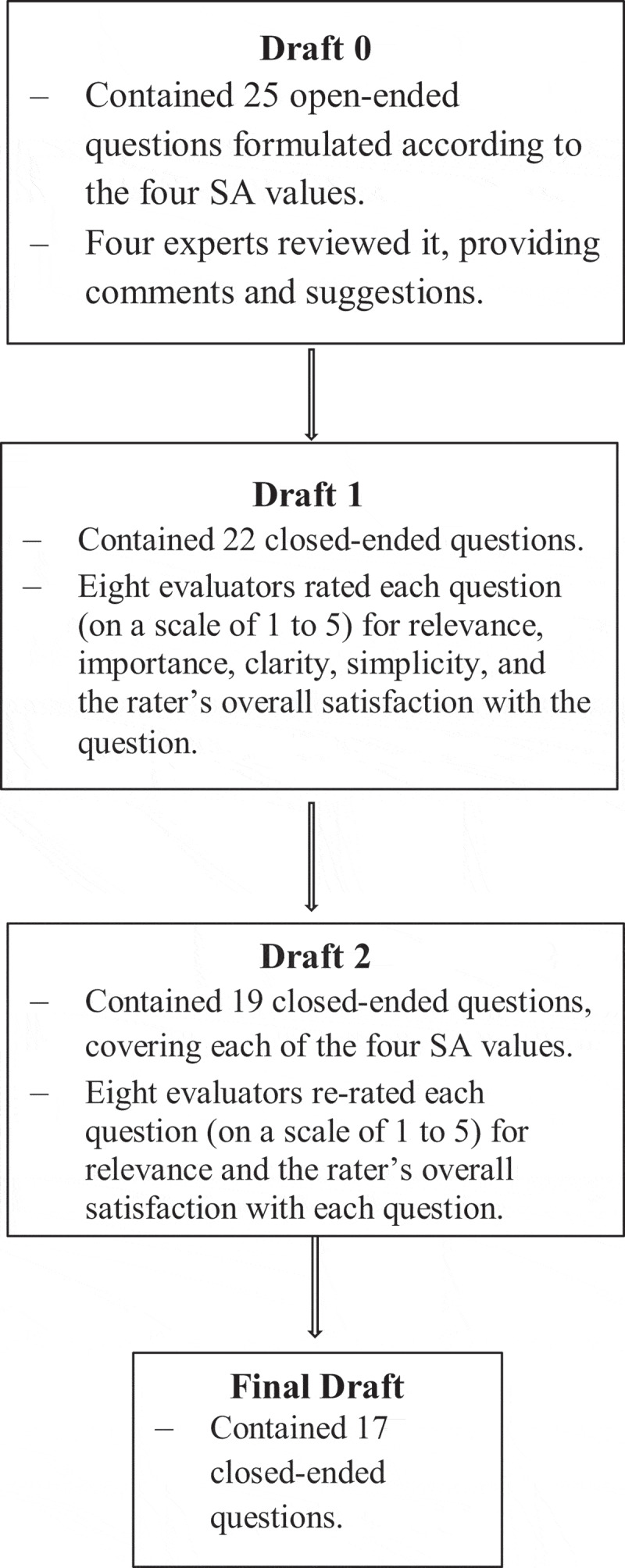
Diagram describing the research methodology and process

These experts were asked to rate each question for relevance, importance, clarity, and simplicity using a 5-point Likert-type scale (1 = not at all; 5 = highest level). They were also asked to provide comments for each open-ended question. The authors added a question after each item, asking the experts to state whether the items were designed in the best possible manner and giving the experts the opportunity to suggest additional or reformulated questions (see Figure 1).

These responses were used to calculate the mean rating for each question. They were then further exploited to determine the level of expert agreement on each item’s relevance by calculating the content validity index (CVI). The following indices were calculated for the CVI: the item-level content validity index (I-CVI), scale-level content validity index average calculation method (SCVI/Ave), and scale-level content validity index universal agreement calculation method (SCVI/UA) [21].

Because this study utilised a minimum of three respondents per question, any item with a CVI greater than or equal to 0.80 was considered excellent [21]. The target value for the SCVI/Ave was also 0.80 or higher [21]. The calculations used the formulae below.

**Table:** 

I-CVI = No. of agreements/total participants
SCVI/Ave = Average of all I-CVIs
SCVI/UA = Total agreements (from I-CVI)/total items

I-CVI = No. of agreements/total participants

SCVI/Ave = Average of all I-CVIs

SCVI/UA = Total agreements (from I-CVI)/total        items

The authors extensively reviewed any item that received comments from the raters or a mean response of less than four on the scale. Based on the experts’ ratings, feedback, and suggestions for Draft 1, three questions were deleted, and the remaining items’ language, annotations, and options were modified to create Draft 2, which contained 19 questions in total.

In round three, Draft 2 was sent to the same eight experts to rate only the relevance of each question and to indicate their overall satisfaction with the inventory on a 5-point Likert-type scale (1 = not satisfied at all; 5 = very satisfied).

Following the third round of analysis, the three CVI indices – I-CVI, SCVI/Ave, and SCVI/UA – were calculated again according to the new ratings given by the experts. Based on these second CVI calculations, two more questions were removed from Draft 2 (because their I-CVI was <0.8) to produce the inventory’s Final Draft, which contained 17 items (see Appendix 1). To test the Final Draft’s validity, the experts were asked to rate the clarity or ambiguity of all the questions. The process of inventory development and validation is shown in the diagram (shown in Figure 1).

## Results

Draft 0 of the inventory contained 25 open-ended questions across four categories. The experts supported the idea of an inventory, their comments referred to the alignment of questions with the four SA values, the structure, language, and complexity of the items. Below are some of their statements.

‘Good idea. You may need more information about the problem: which course, which semester/year, and designed by whom? And is it for PBL, clinical reasoning, or case-based discussion?’

‘This is an important piece of research, especially if your hypothesis is that the PBL scenarios do not support understanding of SA and you need to refashion them.’

‘Most, if not all, the questions are open-ended and must be revised to give the information you need.’

‘Under “Relevance”, you should refer to the “first line of care as a priority intervention in health systems”, because PHC can be interpreted differently and is sometimes is only relevant to under-served areas.’

‘Add a column on the right to collect evidence of answers given and comments.’

For Draft 1 (22 closed-ended questions), the ranges of the mean ratings for the categories assessed by the experts were as follows: relevance, 3.33–4.83; importance, 3.5–4.8; clarity, 3.33–4.83; and simplicity, 3:00–4.67. These characteristics, for some questions, received the highest ratings (i.e. total agreement, 4–5 rating) – 6 for relevance, 10 for importance, 7 for clarity, and 1 for simplicity (see Table 1).

**Table 1.** Number and percentage of total agreements (4–5 rating), Draft 1

Question No.	No. of responders	Relevance		Importance		Clarity		Simplicity	
		No.	%	No.	%	No.	%	No.	%
1	7	6	86	6	86	3	50	4	67
2	6	6	100	5	100	6	100	5	83
3	6	5	83	5	83	4	100	4	67
4	6	6	100	6	100	6	100	6	100
5	6	6	100	6	100	5	83	5	83
6	6	5	83	5	83	6	100	5	83
7	6	6	100	6	100	4	67	4	67
8	6	5	83	3	50	4	67	3	50
9	6	4	67	3	50	4	67	4	67
10	6	2	33	2	33	2	33	2	33
11	6	6	100	6	100	6	100	4	67
12	6	5	83	6	100	5	83	5	83
13	3	2	67	1	33	2	67	2	67
14	3	2	67	3	100	3	100	2	67
15	3	3	100	3	100	2	67	2	67
16	3	2	67	2	67	2	67	2	67
17	3	2	67	2	67	3	100	2	67
18	3	2	67	2	67	1	33	1	33
19	3	2	67	3	100	1	33	1	33
20	3	2	67	2	67	2	67	2	67
21	3	1	33	2	67	2	67	2	67
22	3	2	67	3	100	2	67	1	33

The results for the three CVI indices indicated improvement with each recalculation across the three steps of analysis, except for the SCVI/UA, in which the second and final calculations were equal (Tables 2 and 3).

**Table 2.** I-CVI indices and the agreement number for each question for the first, second, and final rounds of evaluation

SA Value	Question No.	No. of Agreements			I-CVI		
		Draft 1	Draft 2	Final Draft	1^st^ Time	2^nd^ Time	3^rd^ Time
**Relevance**	1	6	5	5	0.86	1	1
	2	6	5	5	1	1	1
	3	5	3	4	0.83	0.8	0.8
	4	6	5	5	1	1	1
	5	6	5	5	1.00	1	1
	6	5	4	4	0.83	0.8	0.8
	7	6	4	4	1.	0.8	0.8
	8	5	1	R2	0.83	0.2	R2
	9	4	4	5	0.67	1	1
	10	2	R1		0.33	R1	
	11	6	5	5	1	1	1
	12	5	5	5	0.83	1	1
**Equity**	13	2	4	4	0.67	0.8	0.8
	14	2	4	4	0.67	0.8	0.8
	15	3	2	4	1	1	0.8
	16	2	4	4	0.67	0.8	0.8
	17	2	4	4	0.67	0.8	0.8
**Cost-Effectiveness**	18	2	2	4	0.67	0.4	0.8
	19	2	R1		0.67	R1	
	20	2	R1		0.67	R1	
**Quality**	21	1	2	R2	0.33	0.66	R2
	22	2	4	4	0.67	0.8	0.8

R1 = Removed after the first rating. R2 = Removed after the second rating.

**Table 3.** First, second, and third SCVI/Ave and SCVI/UA indices for the entire inventory

SCVI/Ave			SCVI/UA		
1^st^ Time	2^nd^ Time	3^rd^ Time	1^st^ Time	2^nd^ Time	3^rd^ Time
0.79	0.88	0.9	0.37	0.47	0.47

Following the first round of rating, Question 10 was merged with Question 9, and Questions 19 and 20 were excluded, because the reviewers agreed that the information they required (relating to cost-effectiveness) would be unlikely to be addressed in problem scenarios. Following the second round of rating, Questions 8 and 21 were removed, because their I-CVI was <0.8. The overall satisfaction with the inventory (satisfied and very satisfied) was 100% for Draft 1 and Draft 2. The final inventory is given in appendix No. 1, all the versions are attached as appendix No. 2.

## Discussion

The development of this inventory was intended to meet the need to include SA in medical programme curricula that requires the employment of different educational strategies. PBL is essential for the application of SA and is a vehicle for education, highlighting various aspects of the social determinants of health and health system studies and improving self-directed learning [15,23]. There is a growing global trend of integrating SA into medical curricula to sensitise learners towards the health disparities, needs, and priorities of the societies they intend to serve. Attempts have been made to apply SA concepts, primarily to educational reform [24–26], but no concrete, measurable tool has been developed to assess the practical applications of this addition [27]. This study aimed to provide information for and develop and test the validity of, an instrument to analyse the application of SA principles in the case scenarios used in PBL medical curricula.

This study used a content validity approach to determine instrument quality [21,28]. Polit and Beck (2006) defined content validity as ‘the degree to which an instrument has an appropriate sample of items for the construct being measured’ [21]. Determining the content validity of an instrument ensures the trust of both readers and researchers since content validity reflects the degree to which a tool covers the content to be analysed [29].

In this study, determining content validity was done through having experts opinion, which is a widespread analytical practice, although it involves simulation-setting judgements [30]. The typical number of experts is generally between three and ten [31,32]; hence, eight experts were invited to participate in this research.

There are various ways to quantify the level of agreement between experts in judging the relevance of content, such as by using the average rating, the coefficient alpha, the kappa coefficient, and similar [21]. This study used CVIs, it is the most widely used method for research in the health professions [21]. One critique of CVIs is that they adjust to chance, unlike kappa statistics. Additionally, CVIs assign expert opinion to only two categories (relevant or not relevant) and concentrate only on the item of relevance, rather than judging the comprehensiveness of the items in adequately measuring the constructs [21]. To account for these shortcomings, the present research included a question asking the experts whether the items in the inventory were formulated accurately and precisely enough while providing them with the opportunity to suggest additional or reformulated questions.

I-CVI was used as the first indicator to refine and revise the inventory items [21], and it showed improvement each time it was applied to the inventory items (a total of three recalculations). For the entire validity index, SCVI/Ave and SCVI/UA were used. Hakek and colleagues reported that S-CVI/Ave is generally preferred; however, they recommended using both indices.

S-CVI/Ave does not depend on the number of raters, but SCVI/UA does, since increasing the number of raters lowers the SCVI/UA. The acceptable values for S-CVI/UA and S-CVI-Ave are 0.8 and above (32). In this study, the SCVI/Ave had a value > 0.8 in the second and third calculations, while the SCVI/UA value was <0.8. This may, perhaps, be explained by the fact that the number of raters in this study was >5.

## Conclusions

Despite the efforts that have been made to translate SA into action, these efforts have mostly focused on defining the dimensions and indices of SA in medical schools. The inventory tool was designed and validated in this study with expectation to help the professional health education schools to translate SA indicators into curricula activities via course design and instructional methods. Moreover, the aim of the inventory to help these schools to identify gaps in their PBL curricula. The targeted gap is, either in the type of case scenarios used or the triggers embedded in the individual case scenarios, which should subsequently lead to SA emphasis through PBL case scenarios that address real societal needs. A revision and rewriting of the problem case scenarios to fit with the incorporation of SA will be the next step.

## Declaration

We declare that

- This research is not involving human subjects
- All data generated or analysed during this study are included in this published article
- Data sharing not applicable to this article as no datasets were generated or analysed during the current study.

## Appendix Appendix No. 1: Final Version of the Inventory

**Analysis of the problem scenarios used in PBL curricula regarding their orientation towards SA principles**

**Problem Title: … … … … … … … … … … … …**

**Unit: … … … … … … … … … … … … … … …**

**Semester/Year: … … … … … … … … … … ……**

- **RELEVANCE**

1. **The problem scenario is relevant to social health concerns.**

(*Social health concerns are the major health problems or health issues in the community (country) as identified by appropriate authorities (local health officials, public health bodies, the Ministry of Health, and/or the community)*).

1. Agree
2. Disagree
3. Neither agree nor disagree
4. Not applicable

(2) **The problem scenario addresses one or more of the social determinants of health.**

(*Social determinants of health are economic and social conditions, and their distributions across the population, influencing individual and group differences in health status, such as income, education, employment, etc*.)

1. Agree
2. Disagree
3. Neither agree nor disagree
4. Not applicable

(3) **The problem scenario points out the relevant principles of health promotion and preventive measures.**

(*Health promotion integrates the three dimensions of the WHO health definition (physical, social, and mental dimensions, and often spiritual health) that result in changed behaviour among individuals and/or the population*).

1. Agree
2. Disagree
3. Neither agree nor disagree
4. Not applicable

(4) **The problem scenario reflects the involvement of different stakeholders in health care.**

(*Stakeholders in health include patients, healthcare providers, insurance providers, family, community, society, government, and non-governmental organisations*).

1. Agree
2. Disagree
3. Neither agree nor disagree
4. Not applicable

(5) **The problem scenario integrates the relevant psychosocial issues, rather than only disease-oriented issues**.
  1. Agree
  2. Disagree
  3. Neither agree nor disagree
  4. Not applicable
(6) **The problem scenario reflects the relevant health system management issues.**

(*Policies and plans adopted by government and other stakeholders to govern and maintain the health of the communities and individuals; e.g. immunisation policy and infectious disease notifications, etc*.)

1. Agree
2. Disagree
3. Neither agree nor disagree
4. Not applicable

(7) **The problem scenario includes the relevant elements of medical professionalism.**

(P*rofessionalism is the competencies or skills expected of a doctor by the public and individual patients; e.g. honesty and integrity*).

1. Agree
2. Disagree
3. Neither agree nor disagree
4. Not applicable

(8) **The problem scenario includes triggers* embedded in the (primary to tertiary) health care referral system based on the case complexity**.
  1. Agree
  2. Disagree
  3. Neither agree nor disagree
  4. Not Applicable
(9) **The problem scenario includes triggers* linked to the evolving roles of doctors in the health system.**

(*The evolving role of doctors in the twenty-first century includes working in teams, utilising resources effectively, providing patient-centred care, advocating for health care systems, and increasing accessibility for patients*).

1. Agree
2. Disagree
3. Neither agree nor disagree
4. Not applicable

(10) **The problem scenario includes triggers* highlighting the importance of a multidisciplinary approach to patient management**.
  1. Agree
  2. Disagree
  3. Neither agree nor disagree
  4. Not applicable

(B) **EQUITY**

(11) **The problem addresses the ethnicity of the patient**.
  1. Yes
  2. No
  3. Not Applicable
(12) **The problem addresses the socioeconomic aspects of the patient**.
  1. Yes
  2. No
  3. Not applicable
(13) **The problem addresses the patient’s age group**.
  1. Yes
  2. No
  3. Not applicable
(14) **The problem addresses the patient’s gender**.
  1. Yes
  2. No
  3. Not applicable
(15) **The problem scenario includes under-served, disadvantaged, or vulnerable populations in society**.
  1. Yes
  2. No
  3. Not applicable

(C) **COST-EFFECTIVENESS**

(16) **The problem scenario includes triggers* for discussing treatment costs and providing alternatives**.
  1. Agree
  2. Disagree
  3. Neither agree nor disagree
  4. Not applicable

(D) **QUALITY**

(17) **The problem scenario includes the concept of ‘person-centred healthcare’.**

(*Person-centred care is a way of thinking and doing things that sees the people who use health as equal partners in planning, developing, and monitoring care to make sure it meets their*

*needs*).

1. Agree
2. Disagree
3. Neither agree nor disagree
4. Not applicable

* PBL triggers are well-mapped educational concepts and strategic clues that lead students to generate learning issues correlated with predetermined curriculum objectives.

## Appendix No. 2: Draft Versions of the Inventory

2–1: Inventory Draft Zero

**Draft Zero: Analysis of the problem’s scenarios in PBL curriculum in relation to their orientation towards social accountability principles**

**Problem Title: … … … … … … … … … … … Unit: … … … … … … … … … … … … … … …**

**Semester/Year: … … … … … … … … … … … Author/s: … … … … … … … … … … … … …**

**Table:**

**SA Values and values related to the GCSA**	**Analysis Points**	**Comment *(Answers should contain explanations)***
Relevance	To what extent does the problem relevant to the society health concerns?	
	What is the evidence of this relevance in the problem scenario?	
	What social determinate of health in the country are pointed in the problem scenario?	
	What principles of health promotion and Preventive measures are pointed in the scenario?	
	What elements of involvement of the society in care and or partnership with stakeholders are pointed in the scenario?	
	What Psycho-social issues are pointed in the scenario?	
	What Health System issues are pointed in the scenario?	
	What element of medical/health professionalism is/are introduced in the scenario?	
	Does the problem include discussion of health care in first line of care as a priority intervention in the health system (e.g PHC) Only?	
	Does the problem include discussion of healthcare in first line of care as a priority intervention in the health system (e.g PHC) and then Referral?	
	Does the problem include discussion of healthcare in Tertiary Level only?	
	Does the problem include discussion of Involvement of Family Medicine (Could be NA)?	
	Does the problem introduce students to the future role of doctors as a change agent in the health system? Please explain through quotes from the scenario	
Equity	The Problem is Addressing which nationality?	
	The Problem is Addressing which social Class?	
	The Problem is Addressing which age group?	
	The Problem is Addressing which gender?	
	The Problem is Addressing underserved, disadvantaged or vulnerable population? Please explain through quotes from the scenario	
Cost-effectiveness	Does the problem include discussion of cost of care? Please explain through quotes from the scenario	
	Does the problem include discussion of cost-effective care? Please explain through quotes from the scenario	
	Are alternatives to high cost interventions be considered? Please explain through quotes from the scenario	
Quality	Does the problem include discussion of quality of Health Care? Please explain through quotes from the scenario	
	Is health care described in the scenario represent a “person-centered healthcare” (Person-centred care is a way of thinking and doing things that sees the people using health and social services as equal partners in planning, developing and monitoring care to make sure it meets their needs.)	
	Are services provided by an integrated health care unit ?, compared to different services disseminated throughout several units.	

2-2 Inventory Draft 1

**Draft 1: Analysis of the problem’s scenarios in the PBL curriculum about their orientation towards social accountability principles**

**Problem Title: … … … … … … … … … … … … … … … … … … … … Unit: … … … … … … … … … … … … … … … … … … …**

**Semester/Year: … … … … … … … … … … … … … … … … … … … Author/s: … … … … … … … … … … … … … … … … …**

**Table:**

**SA Values and values related to the GCSA**	**Analysis Points**	**Comment *(Answers should contain explanations)***
Relevance	To what extent does the problem relevant to the society health concerns?	
	What is the evidence of this relevance in the problem scenario?	
	What social determinate of health in the country are pointed in the problem scenario?	
	What principles of health promotion and Preventive measures are pointed in the scenario?	
	What elements of involvement of the society in care and or partnership with stakeholders are pointed in the scenario?	
	What Psycho-social issues are pointed in the scenario?	
	What Health System issues are pointed in the scenario?	
	What element of medical/health professionalism is/are introduced in the scenario?	
	Does the problem include discussion of health care in first line of care as a priority intervention in the health system (e.g PHC) Only?	
	Does the problem include discussion of healthcare in first line of care as a priority intervention in the health system (e.g PHC) and then Referral?	
	Does the problem include discussion of healthcare in Tertiary Level only?	
	Does the problem include discussion of Involvement of Family Medicine (Could be NA)?	
	Does the problem introduce students to the future role of doctors as a change agent in the health system? Please explain through quotes from the scenario	
Equity	The Problem is Addressing which nationality?	
	The Problem is Addressing which social Class?	
	The Problem is Addressing which age group?	
	The Problem is Addressing which gender?	
	The Problem is Addressing underserved, disadvantaged or vulnerable population? Please explain through quotes from the scenario	
Cost-effectiveness	Does the problem include discussion of cost of care? Please explain through quotes from the scenario	
	Does the problem include discussion of cost-effective care? Please explain through quotes from the scenario	
	Are alternatives to high cost interventions be considered? Please explain through quotes from the scenario	
Quality	Does the problem include discussion of quality of Health Care? Please explain through quotes from the scenario	
	Is health care described in the scenario represent a “person-centered healthcare”	
	(Person-centred care is a way of thinking and doing things that sees the people using health and social services as equal partners in planning, developing and monitoring care to make sure it meets their needs.)	
	Are services provided by an integrated health care unit ?, compared to different services disseminated throughout several units.	

2-2 Inventory Draft 2

**Analysis of the problems scenarios in the PBL curriculum about their orientation towards social accountability principles**

**Problem Title: … … … … … … … … … … … … … … … … … … … … Unit: … … … … … … … … … … … … … … … … … … …**

**Semester/Year: … … … … … … … … … … … … … … … … … … … Author/s: … … … … … … … … … … … … … … … … …**

**RELEVANCE**

1. **The problem is relevant to the social health concerns**

*Social health concerns are the major health problems or health issues in the community (country) as identified by appropriate authorities (MOH, Local Health officials public health bodies and/or the community) e.g. Diabetes, Obesity, etc.*

1. Strongly disagree
2. Disagree
3. Neither agree nor disagree
4. Agree
5. Strongly agree
  (2) **The problem scenario addresses one or more of the social determinates of health**

*Social Determinants of health are the economic and social conditions and their distribution among the population that influence individual and group differences in health status, such as (income, education, employment, etc.*

1. Strongly disagree
2. Disagree
3. Neither agree nor disagree
4. Agree
5. Strongly agree
6. Not applicable
  (3) **The problems scenario points out the relevant principles of health promotion and preventive measures**

*Health promotion integrates the three dimensions of the WHO health definition: physical, social, and mental dimensions (and often, spiritual health) that results in changing behavior among individuals and/or the population*

1. Strongly disagree
2. Disagree
3. Neither agree nor disagree
4. Agree
5. Strongly agree
  (4) **The problem scenario reflects involvement of different stakeholders in health care such as societ, community, population, other governmental sectors, NGOs, the Insurance sector, patients, health institutes etc.**

*Stakeholders in health include the community*,

1. Strongly disagree
2. Disagree
3. Neither agree nor disagree
4. Agree
5. Strongly agree
  (5) **The problem scenario integrates the relevant psycho-social issues and not only a disease-oriented issue**

1. Strongly disagree
2. Disagree
3. Neither agree nor disagree
4. Agree
5. Strongly agree
  (6) **The problem scenario reflects the relevant health system management issues**

*Health System Management: Policies and plans adopted by government and other stakeholders to govern and maintain the health of the communities and individuals e.g.: immunisation policy, referral system, infectious disease notifications, etc*.

1. Strongly disagree
2. Disagree
3. Neither agree nor disagree
4. Agree
5. Strongly agree
  (7) **The problem scenario includes the relevant elements of medical/health professionalism**

*professionalism is the competencies or skills expected of a doctor by the public and individual patients.*

1. Strongly disagree
2. Disagree
3. Neither agree nor disagree
4. Agree
5. Strongly agree
  (8) **The problem scenario includes triggers/cues related to first line of health care (primary health) only**

*PBL trigger is well-mapped educational concepts and strategic clues, thereby lead students to generate learning issues co-related with predetermined curriculum objectives*

1. Strongly disagree
2. Disagree
3. Neither agree nor disagree
4. Agree
5. Strongly agree
  (9) **The problem scenario includes triggers related to referral system in health care (primary to tertiary) based on the case complexity**

1. Strongly disagree
2. Disagree
3. Neither agree nor disagree
4. Agree
5. Strongly agree
6. Not Applicable
  (10) **The problem scenario includes triggers related to the evolving roles of doctors in the health system**

Health care system is defined broadly, to include health care services, organisations and professionals

*Evolving role of doctors in the 21^st^ century are:*

- Work in teams, work more collaboratively
- Utilise resources effectively
- Patient-centred care, or provide care dedicated to patients
- Education of patients on limits of resources etc.
- Advocacy for health care system and primary care
- Advocacy for physicians and health care staff
- Work longer hours and increase accessibility for patients

1. Strongly disagree
2. Disagree
3. Neither agree nor disagree
4. Agree
5. Strongly agree
  (11) **The problem scenario includes triggers to highlight the importance of multidisciplinary approach to patient management**

1. Strongly disagree
2. Disagree
3. Neither agree nor disagree
4. Agree
5. Strongly agree

**EQUITY**

(12) **The problem addresses the ethinicity of the patient?**

1. Yes
2. No
3. Not Applicable
  (13) **The problem addresses the socioeconomic aspect of the patient**

1. High-income class
2. Middle-income class
3. Low-income class
4. Not mentioned
  (14) **The problem addresses the age group**

1. Infancy
2. Children
3. Youth
4. Middle Age
5. Geriatrics
  (15) **The problem addresses the gender of the patient**

1. Male
2. Female
  (16) **The problem scenario includes underserved, disadvantaged or vulnerable population**

1. Strongly disagree
2. Disagree
3. Neither agree nor disagree
4. Agree
5. Strongly agree

**COST-EFFECTIVENESS**

(17) **The problem scenario includes triggers to discuss treatment cost and provide alternatives**

1. Strongly disagree
2. Disagree
3. Neither agree nor disagree
4. Agree
5. Strongly agree

**QUALITY**

(18) **The problem scenario includes triggers related to the discussion of the quality in health care**

*WHO definition of quality of care is ‘the extent to which health care services provided to individuals and patient populations improve desired health outcomes. In order to achieve this, health care must be safe, effective, timely, efficient, equitable and people-centred.’*

1. Strongly disagree
2. Disagree
3. Neither agree nor disagree
4. Agree
5. Strongly agree
  (19) **The problem scenario includes the concept of ‘person-centred healthcare.’**

Person-centred care is a way of thinking and doing things that sees the people using health as equal partners in

*planning, developing and monitoring care to make sure it meets their needs*

1. Strongly disagree
2. Disagree
3. Neither agree nor disagree
4. Agree
5. Strongly agree
